# Genetic polymorphism of glutathione S-transferase P1 (*GSTP1)* in Delhi population and comparison with other global populations^☆^

**DOI:** 10.1016/j.mgene.2013.12.003

**Published:** 2014-01-20

**Authors:** Anita Sharma, Arvind Pandey, Shashi Sharma, Indranil Chatterjee, Ravi Mehrotra, Ashok Sehgal, Joginder K. Sharma

**Affiliations:** a Division of Molecular Diagnostics, Institute of Cytology and Preventive Oncology (ICMR), I-7, sector 39, NOIDA-201301, India; b Centre for Biotechnology, University of Allahabad, Allahabad- 211001, India; c Division of Epidemiology and Biostatistics, India; d Division of Molecular Oncology, Institute of Cytology and Preventive Oncology (ICMR), I-7, NOIDA, India; e Institute of Cytology and Preventive Oncology (ICMR), I-7, Sector 39, NOIDA-201301, Uttar Pradesh, India; f Department of Life Science, Central University of Tamilnadu, Thiruvarur-610101, India

**Keywords:** GST polymorphism, GSTP1, North Indians, ARMS assay, Glutathione S-transferase

## Abstract

Glutathione S-transferases (GSTs) belong to a super family of phase II detoxification enzymes, which play an important role in protecting cells from damage caused by endogenous and exogenous compounds by conjugating reactive intermediates with glutathione to produce less reactive water-soluble compounds. In the present study, we determined the frequencies of two polymorphisms in exon 5 and exon 6 of *GSTP1* gene in 500 normal individuals from Delhi. *GSTP1* polymorphism was analysed by PCR-RFLP using amplification refractory mutation system (ARMS) assay. Two polymorphic sites in *GSTP1* (Ile105 → Val105; Ala114 → Val114) have been analysed simultaneously, which results in four alleles: GSTP1*A (wild-type Ile105; Ala114), GSTP1*B (Val105; Ala114), GSTP1*C (Val105; Val114) and GSTP1*D (Ile105; Val114). The *GSTP1* allele frequency in Delhi population was 0.663, 0.248, 0.069, and 0.020 for GSTP1*A, GSTP1*B, GSTP1*C, and GSTP1*D respectively. The frequency of Ile105 and Val105 allele was 0.683 and 0.317 respectively and it was calculated for the purpose of comparison with published data where all the four alleles were not analysed. *GSTP1* alleles from Delhi population were compared with reported frequencies from all over India, and from other ethnic groups worldwide. This study would provide a basic database for future genetic studies.

## Introduction

Glutathione S-transferases (GSTs), found in virtually all eukaryotes, are a multigene family of phase-II metabolic enzymes, which catalyze the conjugation of reduced glutathione with a variety of endogenous and exogenous electrophilic compounds, including several potentially toxic carcinogens and chemotherapeutic drugs (Hayes et al., 2005), thereby reducing the reactivity of the compounds by making them water soluble and favouring their elimination from the body. In mammals eight classes of GSTs, i.e. alpha (GSTA), mu (GSTM), theta (GSTT), Pi (GSTP), zeta (GSTZ), sigma (GSTS), kappa (GSTK), and omega (GSTO) have been identified (Mannervik et al., 1992), based on sequence homology and substrate specificity. The GSTs have well-established polymorphisms in human populations. The proteins encoded by the different alleles show different abilities to metabolize carcinogens and anticancer agents. It suggests an association between GST polymorphism and the risk for a variety of cancers as well as between said polymorphism and varying responses to cancer treatments. GSTs may also modify susceptibility in certain ethnic groups, showing ethnic dependent polymorphism.

Among all classes of GSTs, *GSTM1*, *GSTT1* and *GSTP1* polymorphisms are extensively studied worldwide. The *GSTM1* and *GSTT1* genes are located on chromosome 1p13.3, and 22q11.2 respectively. Homozygous deletions of *GSTM1* and *GSTT1* genes are common and result in a complete loss of enzyme activity. The frequencies of *GSTM1* null alleles display race and ethnic variations, being highest in Europeans (42–60%) & Asians (41–63%) compared with that of Africans (16–36%) (Cotton et al., 2000, Gao et al., 2010, Hayes et al., 2005, Katoh et al., 2008). However, the frequency of *GSTT1* null genotypes is somewhat less in Europeans (13.31%) compared with that of Africans (14–57%) and in Asians (35–48%) (Cotton et al., 2000, Hayes et al., 2005, Katoh et al., 2008, Sharma et al., 2012).

The glutathione *S*-transferase P1 (*GSTP1*) gene spanning approximately 2.8 kb is located at 11q13 and contains seven exons (Cowell et al., 1988, Kano et al., 1987). Two polymorphic sites in the coding DNA sequence of the *GSTP1* gene have been identified, which are characterized by an A→G transition at nucleotide 313, translating an isoleucine → valine substitution at codon 105 (Ile^105^ → Val^105^) in exon 5 and in second, a C→T transition at nucleotide 341 resulting in replacement of alanine → valine at the amino acid position114 (Ala^114^ → Val^114^) in exon 6. Hence, the human *GSTP1* locus comprises of four different alleles: GSTP1*A (wild type Ile ^105^ → Ala^114^), GSTP1*B (Val^105^ → Ala^114^), GSTP1*C (Val ^105^ → Val ^114^) and GSTP1*D (Ile^105^ → Val ^114^) (Board et al., 1989, Harries et al., 1997, Kano et al., 1987, Watson et al., 1998). GSTP1 plays a central role in the inactivation of toxic and carcinogenenic electrophiles (Hengstler et al., 1998). GST enzyme activity is significantly lower among individuals with 105Val allele due to polymorphism at nucleotide 313 in the *GSTP1* gene. GST genotypes conferring lower enzyme activity may be of advantage for individuals undergoing chemotherapeutic treatment for neoplastic disease because reduced detoxification potentially enhances effectiveness of cytotoxic drugs. Over the past decade, there has been considerable interest in the biological and clinical consequences of the reported GST polymorphisms. Considering the wide variation in the frequency of *GSTP1* alleles in different ethnic populations, we evaluated the distribution of GSTP1 alleles in Delhi population and compared it with the frequency reported in different states of India and other populations worldwide.

## Materials and methods

### Selection of controls

Peripheral blood from 500 normal controls was collected in vials containing EDTA after receiving their informed consent. The controls selected for the study were either normal volunteers from the Institute, or normal healthy individuals visiting with the patients in various hospitals of Delhi. Information on age, sex, smoking and alcohol habits was obtained. The ethical clearance was obtained from our Institute's ethical committee.

### PCR methodology

**Fig. 1:**
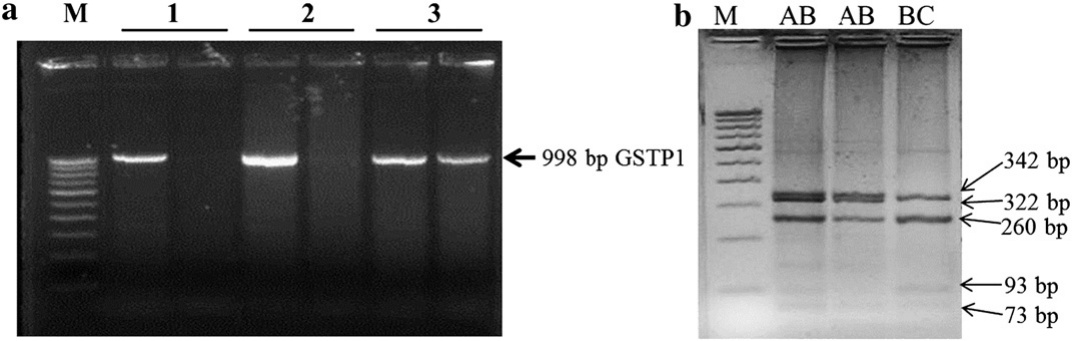
Representative photograph of *GSTP1* gene PCR showing 998 bp band of *GSTP1* and digested PCR product with BsmA1 restriction enzyme and resolved in 3.5% agarose gel using 100 bp ladder marker (M). (a) Samples 1 and 2 showing Ala 114 specific PCR while sample 3 is positive for both Ala 114 and Val114. (b) Digested PCR product showing AB and BC genotype of *GSTP1* gene.

An ARMS (amplification refractory mutation system) assay, described by (Hemmingsen et al., 2001) was performed to identify four alleles of *GSTP1*, using two different sets of primers amplifying exon 5 and exon 6. It included a forward Primer upstream of the codon 105 substitution (5′-ACC CCA GGG CTC TAT GGG AA-3′) and two reverse primers; primer ‘A’ (5′-TCA CAT AGT CAT CCT TGC CGG-3′) (Ala ^114^ specific) and primer ‘B’ (5′-TCA CAT AGT CAT CCT TGC CGA-3) (Val ^114^ specific). Two PCRs were performed for each DNA sample. PCRs were performed in 50 μl reaction volume containing 50–100 ng of genomic DNA, 50 mM KCl, 2.5 mM MgCl_2_, 200 mM Tris (pH 8.4), 200 μM of dNTP each, 1.5 units of Ampli Taq DNA polymerase (Bangalore Genei), forward primer and reverse primers A or B (0.3 μM each). PCR was performed with initial denaturation at 94 °C for 5 min, followed by 35 cycles of denaturation at 94 °C for 1 min, annealing at 62 °C for 1 min, elongation at 72 °C for 2 min, and a final extension at 72 °C for 7 min. A 998 bp fragment was amplified (Fig. 1a).

PCR products were digested with BsmA1 restriction enzyme and resolved in 3.5 % agarose gel. Digested PCR products gave fragments of 73, 260, 322 and 343 bp with Ile ^105^ (GSTP1*A or GSTP1*D) and fragments of 73, 93, 250, 260, and 322 bp with Val ^105^ (GSTP1*B or GSTP1*C) and six fragments of 73, 93, 250, 260, 322, and 343 bp in heterozygote individuals (Ile ^105^/val^105^). The initial ARMS PCR was used to identify Ala^114^ or Val ^114^ genotypes. Therefore, amplification in individuals with Ala ^114^/Ala ^114^ was observed with reverse primer A, and with reverse primer B in individuals having Val ^114^/Val ^114^, and amplification in heterozygote for Ala ^114^/Val ^114^ was observed with both primers A and B. Therefore genotypes in various combinations (AA, AB, AC, BB, AD, BC, BD, CC, CD, and DD) were identified that resulted from A, B, C, and D alleles (Fig. 1b). Most of the studies have reported frequencies of Val and Ile alleles. The frequency of Ile105 and Val105 allele was calculated in the present study for the purpose of comparison with published data where all the four alleles were not analysed.

### Statistical analysis

The data were tabulated and analysed. The mean ± S.D. were computed for quantitative data. The distribution of the allele and genotype frequencies of *GSTP1* was determined by direct counting. Observed frequencies of genotypes in Delhi population were compared with other Indian as well as global populations using Chi square or Fisher's exact test when expected frequencies were small. Hardy–Weinberg equilibrium was calculated in this population and compared with other global populations. *P* value < 0.05 was considered significant.

## Results

A total of 500 healthy individuals, 274 (54.8%) males: 226 (45.2% females participated in this study. The participants had a median age of 32 years, ranging from 18 to 57 years. The mean age of males and females was 33.1 ± 6.7and 32.6 ± 6.8 respectively and the age distribution was not different between both the sexes (*p* = 0.41). Of 500 individuals, 97 (19.4%) were smokers, 126 (26.5%) were alcoholics and 43(8.6%) were betel nut/tobacco chewers. Among the smokers, 85 (87.6%) were males and 12 (12.4%) were females.

Table 1 represents genotype and allele frequencies of *GSTP1* gene polymorphisms in Delhi population. All individuals were analyzed for *GSTP1* polymorphisms and most of them were having *GSTP1*AA (44.6%), *GSTP1* AB (33.8%) and *GSTP1* AC (8.2%) genotypes (Table 1). The number of individuals with other genotypes was very less. Frequencies of four alleles of *GSTP1* namely GSTP1*A, GSTP1*B, GSTP1*C and GSTP1*D were 0.663, 0.248, 0.069 and 0.020 respectively (Table 1). No significant difference in the frequencies of the *GSTP1* gene between males and females, smokers and non-smokers, alcoholics and non- alcoholics, tobacco chewers and non-chewers (*p* > 0.05) was observed (data not shown).The frequency distribution of *GSTP1* alleles in Indian and global populations is tabulated in Table 2, Table 3, Table 4. There are only few studies where the frequency of GSTP1*A, GSTP1*B, GSTP1*C and GSTP1*D was calculated (Table 2). In all these studies the differences in frequencies were not significant except in one study from Egypt, where the frequencies were reversed from that of our observation and the differences were highly significant (*p* < 0.001). In Egyptians, both GSTP1*C and GSTP1*D alleles were not observed. GSTP1*D was absent in two Italian studies and rare in other studies also (Table 2).

In India the frequency of *GSTP1* Ile105/Ile105 allele was almost similar throughout India (*p* = 0.05) with a few exceptions (Bid et al., 2010, Bose and Bathri, 2012, Dunna et al., 2012, Konwar et al., 2010, Qadri et al., 2011, Vettriselvi et al., 2006, Vijayalakshmi et al., 2005), where the frequency was high. In one of the studies from Jammu & Kashmir (Qadri et al., 2011) the frequency of *GSTP1* Ile105/Ile105 allele was so high as compared with the present study, that after including this single study the differences became significant (*p* < 0.001) (Table 3). The frequencies of *GSTP1* genotypes did not achieve Hardy–Weinberg equilibrium in the clubbed data of North Indians (*p* < 0.001), South Indians (*p* < 0.002) and Central Indians (*p* < 0.01) (Table 3).

We have calculated and compared the frequency of *GSTP1* alleles in four main world populations namely Africans, Europeans, Asians and Indians, with the present study (Table 4). The gene frequency was obtained by clubbing the individual data from various populations. The mean gene frequency of Ile/Val allele was (0.657/0.343; *N* = 1606) in Africans, (0.686/0.314; *N* = 4655) in Europeans, (0.779/0.221; *N* = 3486) in Asians and (0.719/0.281; *N* = 3035) in Indians. Genotype frequencies of *GSTP1* Ile105Val polymorphism followed Hardy–Weinberg equilibrium in Europeans (*p* = 0.62), and Asians (*p* = 0.77) but not in Africans (*p* = 0.004) and Indians (*p* < 0.001). The frequency of Ile105 and Val105 allele was observed to be 0.683 and 0.317 respectively in the present study and the population was in Hardy–Weinberg equilibrium. Highly significant (*p* < 0.0001, df = 1, χ^2^ = 625.87) linkage disequilibrium was observed between Ile105 and Ala114 and between Val105 and Val114.

## Discussion

GSTs are involved in the biotransformation of exogenous substances, including mutagens, carcinogens, and other poisonous chemicals, and play a crucial role in the detoxification process, thereby protecting cells from these compounds (Strange et al., 2001). Epidemiological studies have suggested that individuals differing in the expression of allelic variants of *GSTP1* gene differ in susceptibility to various chemical carcinogens (Nakajima and Aoyama, 2000). In the present study we have examined the *GSTP1* Ile/Val polymorphism by ARMS assay to know the base data of GSTP1*A, GSTP1*B, GSTP1*C and GSTP1*D alleles in normal Delhi population. The information on frequencies of these four alleles was available in a few studies (Table 2). They have also observed almost similar results and the differences in frequencies were not statistically significant when compared with the present study (Ballerini et al., 2003, Bernardini et al., 2005, Hemmingsen et al., 2001). However in one study from Egypt, statistically significant differences emerged. The reason for this variation is not known, may be the number of individuals was very less in that study (Abdel-Alim et al., 2007).

The frequency distribution of GSTP1 alleles observed in the present study is almost similar throughout India except for Jammu & Kashmir, Lucknow, Central India and South India, thus suggesting a distinct difference among the Indian population. The reason for this diversity in the Indian population is believed to be because of different socio-cultural traditions, lifestyles and exposures.

Distinct ethnic differences also exist in the frequency of *GSTP1* Ile/Val polymorphism. The range of Ile allele frequency was 0.47–0.86 in Africans, 0.63–0.76 in Europeans, 0.67–0.92 in Asians and 0.59–0.84 in Indians (from data mentioned in Table 4). The frequency distribution of mutant allele *Val105/Val105* was most frequent among South African Xhosas (0.53) and African American (0.51) and least frequent among Tanzanians (0.14) (Adams et al., 2003, Dandara et al., 2002, Millikan et al., 2000). The range of *Val105/Val105* allele was 0.14–0.53 in Africans, 0.23–0.37 in Caucasians, 0.08–0.33 in Asians and 0.16–0.41 in Indians (from data mentioned in Table 4). *GSTP1 105Val* is less frequent in Asians as compared to Caucasians. A complete absence of the mutant homozygous *Val105/Val105* genotype was also reported in one of the studies from Japan (Kiyohara et al., 2003). Observation from the present study of the genotype distribution of *GSTP1* Ile/Ile and Ile/Val reveals that it varies significantly from Asians (*p* < 0.0001) but not from Africans, Europeans and Indians. Similar results were also observed in other studies (Mishra et al., 2004, Polimanti et al., 2011, Tan et al., 2000).

The *GSTP1* Val/Val genotype is uncommon and exists in 5% of Caucasians (Garte et al., 2001). This form of the *GSTP1* enzyme has been reported to be 2–3 times less stable than the Ile105 form (Johansson et al., 1998) and possibly associated with a higher level of DNA adducts (Ryberg et al., 1997). Individuals with *GSTP1* Val/Val genotype had significantly better survival in hepatocellular carcinoma patients (Cheng-Gang et al., 2012), but this genotype is associated with worse outcome in basal cell carcinoma (Ramachamdran et al., 2000) and breast cancer (Zhang et al., 2011). In a meta analysis it has been reported that individuals with *GSTP1* Ile105/Val 105 genotype increase susceptibility to breast cancer in Asian population (Lu et al., 2011), while a good response and light toxicity were also observed in breast cancer patients carrying *GSTP1* Ile105/Val 105 or Ile 105/Ile105 genotype (Zhang et al., 2011). Individuals with homozygous genotypes of *GSTP1* Ile/Ile were reported to have the lowest risk of prostate cancer (Kote-Jarai et al., 2001), and this genotype is found to be protective in rheumatoid arthritis (Mattey et al., 1999) and basal cell carcinoma (Ramachamdran et al., 2000). An over expression of GSTP1 enzyme in individuals with Ile/Ile genotype causes resistance to drugs like cisplatin in oral and maxillofacial carcinoma (Townsend and Tew., 2003, Xu et al., 2002). Thus GST polymorphism studies will provide a clue to the identification of responders to cancer therapy with certain chemotherapeutic drugs.

GSTP1 associated risk is probably disease-dependent, and may reflect differences in relevant substrates. Individuals with different combinations of *GST* alleles would also help in studying the effect of various carcinogens in different populations having various exposures and giving personalised treatment in case of cancer. Understanding the contribution of *GST* gene polymorphisms and their interactions with other relevant factors may improve screening diagnostic assays for various cancers. Thus, our results signify the impact of ethnicity and provide a basis for future epidemiological and clinical studies.

## Figures and Tables

**Table 1:** *GSTP1* genotype and allele frequency in Delhi population.

Genotype	AA	AB	BB	AC	AD	BC	BD	CC	CD	DD
No. of individuals (*N* = 500)	223	169	30	41	7	17	2	2	7	2
(44.6%)	(33.8%)	(6.0%)	(8.2%)	(1.4%)	(3.4%)	(0.4%)	(0.4%)	(1.4%)	(0.4%)


Total number of alleles corresponding to above genotypes											Total alleles	Allele frequency
GSTP1*A alleles	446	169		41	7						663	0.663
GSTP1*B alleles		169	60			17	2				248	0.248
GSTP1*C alleles				41		17		4	7		69	0.069
GSTP1*D alleles					7		2		7	4	20	0.020

**Table 2:** Distribution of *GSTP1* genotypes and allele frequency by ARMS assay in various populations.

Population	No. of individuals	*GSTP1*⁎A	*GSTP1*⁎B	*GSTP1*⁎C	*GSTP1*⁎D	*P* value	Reference
No.	No.	No.	No.
(%; frequency)	(%; frequency)	(%; frequency)	(%; frequency)
European	58	33	17	7	1	P = 0.41	(Hemmingsen et al., 2001)
(56.9; 0.57)	(29.3; 0.30)	(12.1; 0.12)	(1.7; 0.01)
Italian	250	177	60	13	0	*P* = 0.09	(Ballerini et al., 2003)
(70.8; 0.71)	(24.0; 0.24)	(5.2; 0.05)	0
Italian	228	164	52	12	0	*P* = 0.1	(Bernardini et al., 2005)
(71.9; 0.72)	(22.8; 0.23)	(5.26; 0.05)	0
Egyptian	40	10	30	0	0	*P* < 0.001⁎	(Abdel-Alim et al., 2007)
(25.0; 0.25)	(75.0; 0.75)	0	0
Delhi	500	331	124	35	10	Reference	Present study
(66.2; 0.66)	(24.8; 0.25)	(7.0; 0.07)	(2.0; 0.02)

⁎ Significant.

**Table 3:** Genotype and allele frequencies of *GSTP1* in various Indian populations.

Populations	Total number	Genotype			Allele		*P* value	References
Ile/Ile	Ile/Val	Val/Val	Ile	Val
No	No	No	Frequency	
North Indians	1179	596	531	52	0.731	0.269	*P* = 0.05	(Bid et al., 2010, Konwar et al., 2010, Mishra et al., 2004, Qadri et al., 2011, Soya et al., 2005, Srivastava et al., 2005)
(50.6%)	(45.0%)	(4.40%)
South Indians	1448	720	635	93	0.717	0.283	*P* = 0.11	(Dunna et al., 2012, Lakkakula et al., 2013, Samson et al., 2007, Soya et al., 2005, Vettriselvi et al., 2006, Vijayalakshmi et al., 2005)
(49.72%)	(43.86%)	(6.42%)
Central Indians	40	18	12	10	0.60	0.40	*P* = 0.004⁎	(Bose and Bathri, 2012)
(44.0%)	(30.0%)	(26.0%)
Present study	500	225	233	42	0.683	0.317	Reference	Present study
(45.0%)	(46.60%)	(8.40%)

⁎ Significant, except the study done by Qadri et al., 2011.

**Table 4:** Genotype and allele frequencies of *GSTP1* in Worldwide populations.

Population	Total no.	Genotype			Allele frequency		*P* value	References
Ile/Ile	Ile/Val	Val/Val	Ile	Val
No.	No.	No.
Africans	1606	725 (45.14%)	660 (41.09%)	221 (13.77%)	0.657	0.343	*P* = 0.33	(Adams et al., 2003, Burim et al., 2004, Dandara et al., 2002, Masimirembwa et al., 1998, Millikan et al., 2000, Rossini et al., 2002, Watson et al., 1998)
Europeans	4655	2196 (47.18%)	1992 (42.79%)	467 (10.03%)	0.686	0.314	*P* = 0.90	(Agalliu et al., 2006, Andonova et al., 2010, Fontana et al., 2009, Gsur et al., 2001, Harries et al., 1997, Jerónimo et al., 2002, Kote-Jarai et al., 2001, Loktionov et al., 2001, Millikan et al., 2000, Mitrunen et al., 2001, Shepard et al., 2000, Sørensen et al., 2007, Soya et al., 2005, Steinhoff et al., 2000, Vlaykova et al., 2012, Watson et al., 1998, Welfare et al., 1999)
Asians	3486	2119 (60.79%)	1194 (34.25%)	173 (4.96%)	0.779	0.221	*P* < 0.0001*	(Ansari et al., 2010, Aynacioglu et al., 2004, Cheng-Gang et al., 2012, Cho et al., 2005, Chonlada et al., 2009, Kawai et al., 2005, Kiran et al., 2010, Kiyohara et al., 2003, Kunak et al., 2012, Lee et al., 2005, Soya et al., 2005, Watson et al., 1998)
Indians	3035	1518 (50.01%)	1329 (44.12%)	188 (5.87%)	0.719	0.281	*P* = 0.10	(Bid et al., 2010, Bose and Bathri, 2012, Dunna et al., 2012, Konwar et al., 2010, Mishra et al., 2004, Qadri et al., 2011, Samson et al., 2007, Soya et al., 2005, Soya et al., 2007, Srivastava et al., 2005, Vettriselvi et al., 2006, Vijayalakshmi et al., 2005)
Present study	500	225 (45.0%)	233 (46.6%)	42 (8.4%)	0.683	0.317	Reference	

## Appendix A Supplementary data

Table 5 Allele and genotype frequencies of GSTP1 worldwide.
