# Supplementary material for: Genetic polymorphism of glutathione S-transferase P1 (GSTP1) in Delhi population and comparison with other global populations
Source: Meta Gene. 2014 Jan 20;2:134–42. doi: 10.1016/j.mgene.2013.12.003 (PMC4287809; doi:10.1016/j.mgene.2013.12.003)
Supplement: Table 5 — Allele and genotype frequencies of GSTP1 worldwide. [file mmc1.pdf]

Supplementary data:

Table 5: Allele and Genotype frequencies of GSTP1 Worldwide

**Population**		Genotype No. (%)				Allele Frequencies		Reference
**Africans**	No. Of subjects	Ile/Ile	Ile/Val	Val/Val	P value	Ile	Val	
Brazilian (White)	319	164 (51.4)	109 (34.2)	46 (14.4)	P<0.001*	0.69	0.31	(Rossini et al., 2002)
Brazilian (non-white)	272	130 (47.8)	116 (42.6)	26 (9.6)	P=0.56	0.69	0.31	(Rossini et al., 2002)
North Carolina (African American)	137	48 (35.0)	63 (46.0)	26 (19.0)	P<0.001*	0.58	0.42	(Watson et al., 1998)
African American	271	60 (22.0)	149 (55.0)	62 (23.0)	P<0.001*	0.49	0.51	(Millikan et al., 2000)
South African Xhosa	101	22 (22.0)	51 (50.0)	28 (28.0)	P<0.001*	0.47	0.53	(Adams et al., 2003)
Zimbabwean	97	56 (58.0)	35 (36.0)	6 (6.0)	P=0.08	0.76	0.24	(Masimirembwa et al., 1998)
Venda	86	66 (77.0)	12 (14.0)	8 (9.0)	P<0.001*	0.84	0.16	(Masimirembwa et al., 1998)
Brazil	221	100 (45.3)	103 (46.6)	18 (8.1)	P=0.99	0.68	0.32	(Burim et al., 2004)
Tanzanians	102	79 (77.0)	22 (22.0)	1 (1.0)	P<0.001*	0.86	0.14	(Dandara et al., 2002)
**Europeans**								
White (USA)	392	157 (40.0)	192 (49.0)	43 (11.0)	P=0.22	0.64	0.36	(Millikan et al., 2000)
Euro American	287	121	146	20	P=0.47	0.67	0.33	(Watson et

		(42.0)	(51.0)	(7.0)				al., 1998)
Europe	803	365 (45.4)	354 (44.1)	84 (10.5)	P=0.41	0.66	0.34	(Shepard et al., 2000)
New Castle UK, (Caucasians)	178	80 (44.9)	77 (43.4)	21 (11.7)	P=0.38	0.67	0.33	(Welfare et al., 1999)
Caucasians	166	65 (39.2)	79 (47.3)	22 (13.3)	P=0.13	0.63	0.37	(Gsur et al., 2001)
Finnish (Caucasians)	481	266 (55.3)	181 (37.6)	34 (7.1)	P<0.005*	0.74	0.26	(Mitrunen et al., 2001)
Bulgaria (Caucasians)	126	68 (54.0)	49 (39.0)	9 (7.0)	P=0.21	0.73	0.27	(Vlaykova et al., 2012)
Bulgaria (Caucasians)	104	49 (47.0)	39 (38.0)	16 (15.0)	P<0.05*	0.66	0.34	(Andonova et al., 2010)
Scotland, UK (Caucasians)	155	79 (51)	66 (42.5)	10 (6.5)	P=0.39	0.72	0.28	(Harries et al., 1997)
Surrey, UK (Caucasians)	280	143 (51.2)	108 (38.5)	29 (10.3)	P=0.09	0.70	0.30	(Kote-Jarai et al., 2001)
East Anglia, UK (Caucasians)	355	142 (40)	174 (49)	39 (11)	P=0.23	0.65	0.34	(Loktionov et al., 2001)
Germany, (Caucasians)	100	55 (55.0)	36 (36.0)	9 (9.0)	P=0.14	0.73	0.27	(Steinhoff et al., 2000)
Sweden (Caucasians)	767	376 (49)	307 (40)	84 (11)	P<0.005*	0.69	0.31	(Sørensen et al., 2007)
Portugal (Caucasians)	43	19 (43.3)	20 (47.5)	4 (9.2)	P=0.94	0.67	0.33	Jeronimo et al., 2002
American non-Hispanic (Caucasians)	100	43 (43.0)	46 (46.0)	11 (11.0)	P=0.70	0.66	0.34	(Agalliu et al., 2006)
Caucasians	273	140 (51.3)	105 (38.5)	28 (10.2)	P=0.09	0.71	0.29	(Soya et al., 2005)
French	45	28 (62.0)	13 (29.0)	4 (9.0)	P=0.06	0.76	0.23	(Fontana et al., 2009)

**Asians**								
Japanese	88	62 (70.5)	26 (29.5)	0	P<0.001*	0.85	0.15	(Kiyohara et al., 2003)
Japanese	475	270 (56.8)	181 (38.1)	24 (5.1)	P<0.001*	0.76	0.24	(Kawai et al., 2005)
Chinese	150	91 (60.7)	53 (35.3)	6 (4.0)	P<0.002*	0.83	0.17	(Soya et al., 2005)
Chinese	481	211 (43.9)	220 (45.7)	50 (10.4)	P=0.56	0.67	0.33	(Cheng-Gang et al., 2012)
Korean	1030	704 (68.4)	300 (29.1)	26 (2.5)	P<0.001*	0.74	0.26	(Cho et al., 2005)
Taiwanese	116	78 (67.0)	35 (30.0)	3 (3.0)	P<0.001*	0.82	0.18	(Watson et al., 1998)
Taiwanese	184	112 (60.9)	64 (34.8)	8 (4.3)	P<0.001*	0.78	0.22	(Lee et al., 2005)
Thais	280	154 (55.0)	109 (38.9)	17 (6.1)	P<0.02*	0.77	0.23	(Chonlada et al., 2009)
Iranians Tehran	65	25 (38.5)	38 (58.5)	2 (3.0)	P=0.11	0.68	0.32	(Ansari et al., 2010)
Turkish	265	134 (50.5)	99 (37.4)	32 (12.1)	P<0.03*	0.69	0.31	(Aynacioglu et al., 2004)
Turkish	50	22 (44.0)	26 (52.0)	2 (4.0)	P=0.59	0.70	0.30	(Kiran et al., 2010)
Turkish	302	256 (84.8)	43 (14.2)	3 (1.0)	P<0.001*	0.92	0.08	(Kunak et al., 2012)
**Indian**								
Kashmiri (Jammu & Kashmir)	80	59 (73.7)	17 (21.3)	4 (5.0)	P<0.001*	0.84	0.16	(Qadri et al., 2011)
North Indians	370	164 (44.3)	186 (50.3)	20 (5.4)	P=0.19	0.69	0.30	(Soya et al., 2005)

North Indians	99	57 (57.6)	39 (38.9)	3 (3.5)	P=0.03	0.77	0.23	(Srivastava et al., 2005)
North Indians (Lucknow)	310	137 (44.3)	156 (50.3)	17 (5.4)	P=0.24	0.69	0.31	(Mishra et al., 2004)
North Indians (Lucknow)	200	119 (59.5)	75 (37.5)	6 (3.0)	P<0.001*	0.78	0.22	(Bid et al., 2010)
North Indians (Lucknow)	200	119 (59.5)	75 (37.5)	6 (3.0)	P<0.001*	0.78	0.22	(Konwar et al., 2010)
Central Indians Bhopal	40	18 (44)	12 (30)	10 (26)	P<0.002*	0.59	0.41	(Bose and Bathri, 2012)
South Indians (Tamilnadu)	133	58 (43.6)	63 (47.4)	12 (9.0)	P=0.95	0.67	0.33	(Soya et al., 2005)
South Indians (Chennai)	500	230 (46.0)	219 (43.8)	51 (10.2)	P=0.51	0.68	0.32	(Samson et al., 2007)
South Indians (Chennai)	255	149 (58.5)	98 (38.4)	8 (3.1)	P<0.001*	0.78	0.22	(Vettriselvi et al., 2006)
South Indians (Chennai)	100	43 (43.0)	51 (51.0)	6 (6.0)	P=0.60	0.68	0.31	(Vijayalakshmi, 2005)
South Indians (Hyderabad)	248	140 (56.5)	105 (42.3)	3 (1.2)	p<0.001*	0.78	0.22	(Dunna et al., 2012)
South Indians(Andhra Pradesh)	212	100 (47.2)	99 (46.7)	13 (6.1)	P=0.56	0.70	0.29	(Lakkakula et al., 2013)
North Indians (Delhi)	500	225 (45.0)	233 (46.6)	42 (8.4)	Reference	0.683	0.317	Present study

*Significant
